# Mitochondrial transfer in acute myeloid leukaemia and multiple myeloma: Mechanisms, consequences and potential therapeutic opportunities

**DOI:** 10.1111/febs.70544

**Published:** 2026-04-10

**Authors:** Ebubechukwu Nwarunma, Mark T. S. Williams

**Affiliations:** ^1^ Research Centre for Health, School of Health and Life Sciences, Department of Biological and Biomedical Sciences Glasgow Caledonian University UK; ^2^ School of Cancer Sciences College of Medical, Veterinary and Life Sciences, University of Glasgow UK

**Keywords:** acute myeloid leukaemia (AML), bone marrow microenvironment (BMME), chemoresistance, Horizontal mitochondrial transfer (HMT), Multiple myeloma (MM)

## Abstract

Haematological malignancies, such as acute myeloid leukaemia (AML) and multiple myeloma (MM), which develop from malignant transformations within the bone marrow, represent the most critical unmet needs in the haemato‐oncology field. Sub‐optimal clinical outcomes in patients with AML and MM are often driven by resistance to chemotherapy. It is well established that cells within the bone marrow microenvironment (BMME) support the proliferation and survival of these blood cancer cells. One of the mechanisms by which these BMME‐resident cells support the malignant cells is through horizontal mitochondrial transfer (HMT), a mechanism well documented as occurring under steady‐state conditions as well as in many cancers. Recent research implicates mitochondrial transfer in BMME‐driven chemoresistance in AML and MM. In this review, we critically analyse current understanding of the role of HMT in supporting the survival and proliferation of AML and MM cells, as well as driving resistance to cytotoxic effects of chemotherapy. We further elucidate various mechanisms, molecular triggers, functional consequences, and therapeutic implications for HMT in AML and MM. Our review also highlights unanswered questions within the HMT field and provides a theoretical basis for further study, giving direction on what is important in translating this knowledge into effective future therapeutic strategies.

## Graphical abstract

Mitochondria can be transferred from bone marrow cells to cancer cells in acute myeloid leukaemia and multiple myeloma, boosting tumour energy production, growth, and drug resistance. This review highlights key transfer mechanisms and shows how targeting mitochondrial movement and dynamics may offer new therapeutic strategies to limit cancer progression and improve treatment outcomes.

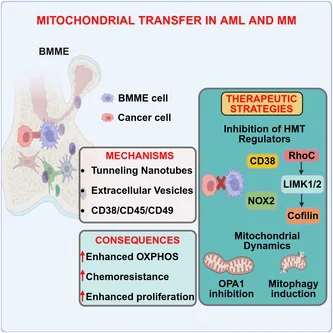

**Abbreviations**
Akt: Protein Kinase B
AML: acute myeloid leukaemia
AMPK: AMP‐activated protein kinase
Ara‐C: cytarabine
ATP: adenosine triphosphate
BCL2: B‐cell lymphoma 2
BCMA: B‐cell maturation antigen
BMME: bone marrow microenvironment
BMSCs: bone marrow stromal cells
CAMKII: calcium/calmodulin‐dependent protein kinase II
CD38: cluster of differentiation 38
Cx43: connexin 43
DNR: daunorubicin
DPI: diphenyleneiodonium
ER: endoplasmic reticulum
EVs: extracellular vesicles
GJs: gap junctions
HSCs: haematopoietic stem cells
Hsp70: heat shock protein 70
IP_3_
: inositol triphosphate
LIMK: LIM domain kinase
LSCs: leukaemic stem cells
LST1: leukocyte‐specific transcript 1
MLL: mixed lineage leukaemia
MM: multiple myeloma
MSC: mesenchymal stem cell
mtDNA: mitochondrial DNA
mTOR: mechanistic target of rapamycin
Myo10: myosin 10
NAD^+^
: nicotinamide adenine dinucleotide (oxidized form)
NADPH: nicotinamide adenine dinucleotide phosphate (reduced form)
NOX2: NADPH oxidase 2
NRF1: nuclear respiratory factor 1
NSG: NOD‐scid IL2rγnull
OXPHOS: oxidative phosphorylation
PGC‐1α: peroxisome proliferator‐activated receptor gamma coactivator 1‐alpha
PI3K: phosphoinositide 3‐Kinase
PINK1: PTEN‐induced putative kinase 1
PP2A: protein phosphatase 2A
RhoC: Ras homolog gene family, member C
ROS: reactive oxygen species
SQSTM1: sequestosome 1
STAT3: signal transducer and activator of transcription 3
TILs: tumour‐infiltrating lymphocytes
TNFAIP2: tumourtumor necrosis factor alpha‐induced protein 2
TNTs: tunnelling nanotubes

## Introduction

**Fig. 1:**
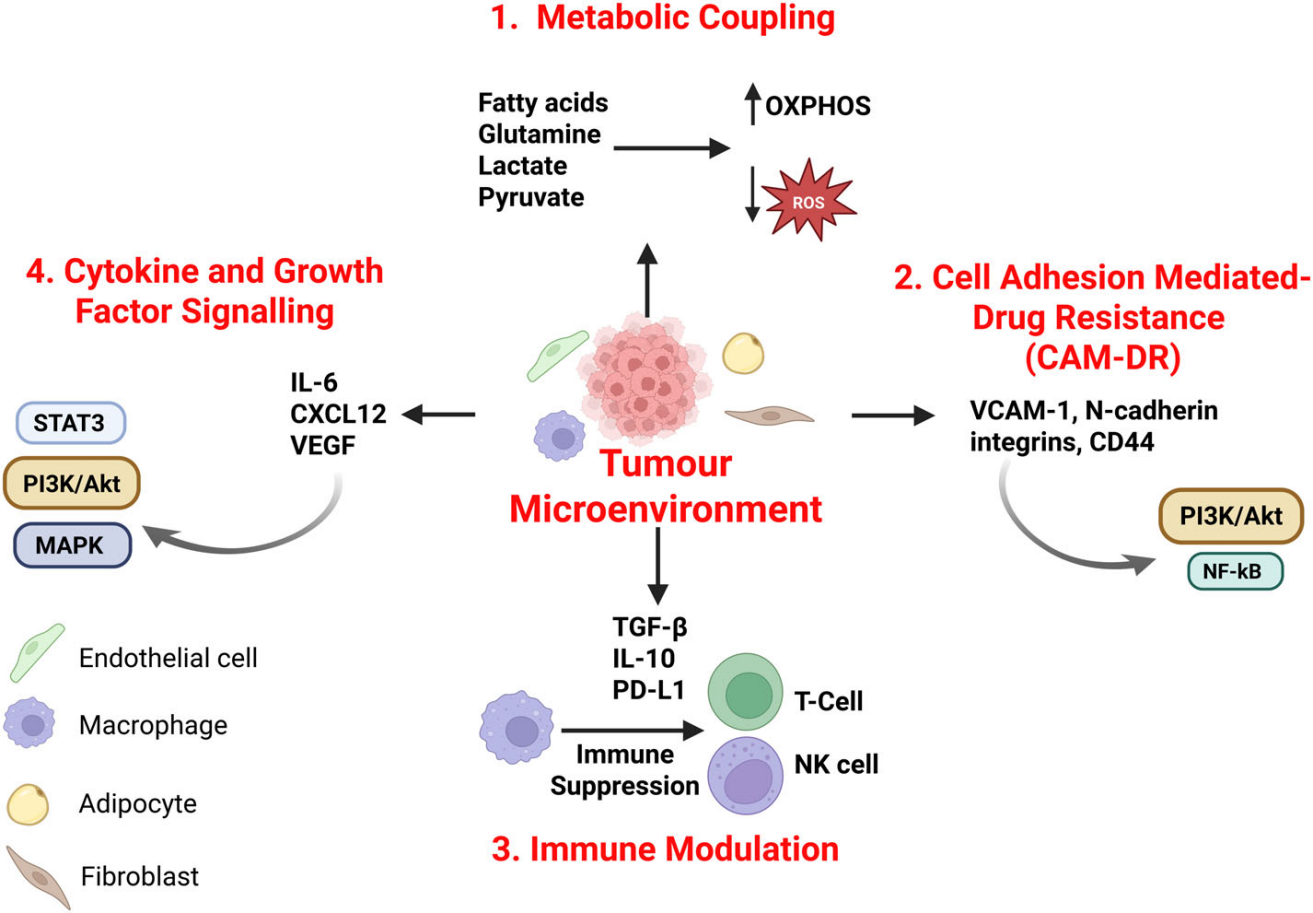
Mechanisms by which resident cells within the bone marrow microenvironment (BMME) support acute myeloid leukaemia (AML) and multiple myeloma (MM) cells. Bone marrow stromal, endothelial, adipocytic, and immune cells provide multifaceted support to AML and MM cells via a variety of mechanisms including metabolic coupling (1), cell adhesion‐mediated‐drug resistance (CAM‐DR) (2), immune modulation (3) and cytokine and growth factor signalling (4). Collectively, these mechanisms establish a metabolically and immunologically protective niche that enhances survival, proliferation, and chemoresistance of AML and MM cells within the bone marrow microenvironment. Created in BioRender. Nwarunma, E. (2026) https://BioRender.com/3hhlajj. Licence agreement number: KN29IK1PWP.

Haematological malignancies, including acute myeloid leukaemia (AML) and multiple myeloma (MM), arise from malignant transformations within the bone marrow haematopoietic myeloid and lymphoid cell compartments respectively. Despite substantial therapeutic advances, relapse and chemoresistance remain pervasive obstacles, often driving poor/sub‐optimal clinical outcomes. Findings from studies over the last two decades have transformed our understanding of the bone marrow microenvironment (BMME), revealing that malignant cells exploit stromal, immune and vascular elements to establish a supportive tumour niche/microenvironment. As a result, the cells resident within the BMME, such as bone marrow stromal cells (BMSCs), fibroblasts, mesenchymal stem cells (MSCs) and immune cells, including macrophages, have been shown to actively support cancer cells via important cell‐to‐cell and non‐cell‐to‐cell contact interactions (Fig. 1). Such interactions involve metabolic coupling where stromal cells are involved in mediating metabolic crosstalk via the supply of key metabolites, such as fatty acids, glutamine, lactate and pyruvate, sustaining oxidative metabolism and redox homeostasis in tumour cells [1]. Stromal cells also interact with other cells within the BMME via cytokine and growth factor signalling where they secrete factors, such as interleukin‐6 (IL‐6), C‐X‐C motif chemokine ligand 12 (CXCL12), and vascular endothelial growth factor (VEGF). These factors activate pro‐survival signalling pathways, including signal transducer and activator of transcription 3 (STAT3) and phosphoinositide 3‐kinase/Akt (PI3K/Akt) [2, 3]. They also drive cell adhesion‐mediated drug resistance (CAM‐DR) via interactions involving integrins, N‐cadherin, vascular cell adhesion molecule 1 (VCAM‐1), and cluster of differentiation 44 (CD44), reinforcing anti‐apoptotic signalling and quiescence of leukaemic stem cells [1, 3, 4]. Stromal cells and macrophages have been shown to drive immune modulation by secreting transforming growth factor β (TGF‐β), interleukin 10 (IL‐10), and expressing programmed death‐ligand 1 (PD‐L1), thereby promoting immune evasion and tumour persistence. In addition, stromal cells and macrophages have been shown to transfer mitochondria to cancer cells [5, 6].

The concept of intercellular mitochondrial transfer has rapidly gained traction as a fundamental mechanism of cellular adaptation. The first seminal scientific demonstration came from Rustom *et al*. (2004), who described tunnelling nanotubes (TNTs) as novel actin‐based structures mediating direct cell‐to‐cell transfer of organelles, including mitochondria [7]. This was followed by the landmark study by Spees *et al*. (2006), who showed that functional mitochondria were transferred from MSCs to mitochondria‐deficient cells, rescuing aerobic respiration in the acceptor [8]. Soon after, Islam *et al*. (2012) demonstrated HMT from BMSCs to alveolar epithelial cells *in vivo*, restoring bioenergetics and improving survival in a mouse model of acute lung injury [9]. The pioneering studies established HMT as both a structural and functional phenomenon, laying the foundation for later work in oncology, including its role in therapy resistance in haematological malignancies.

Mitochondrial transfer has been increasingly recognized as a mechanism by which BMSCs, a primary component of the BMME, donate functional mitochondria to cancer cells via TNTs [10], extracellular vesicles (EVs) [10, 11] and occasionally via gap junctions or other contact‐mediated processes, such as cell fusion [12, 13, 14]. This phenomenon enables enhanced oxidative phosphorylation (OXPHOS) capacity within acceptor cells, increasing ATP production [13], and enabling better energy regulation and the ability of acceptor cells to suppress reactive oxygen species (ROS) [15]. Overall, these functions of HMT contribute to the survival of cancer cells under chemotherapeutic stress [12, 14].

The aim of this review is to evaluate current knowledge of HMT in AML and MM, delineating mechanisms of intercellular communication, functional metabolic consequences, and emerging therapeutic interventions. We will critically examine how mitochondrial acquisition shapes disease progression and relapse, identify potential molecular targets and discuss translational opportunities. By highlighting the role of intercellular mitochondrial dynamics in these malignancies, we seek to advance the development of novel strategies, with the goal of contributing to improvements in clinical outcomes in AML and MM patients. Terminology in the mitochondrial transfer field has been inconsistent; therefore, throughout this review we adopt the consensus nomenclature and characterization principles proposed in the recent consortium statement. For example, use of ‘donor’ and ‘acceptor’ cells, and route‐based descriptors for contact‐dependent versus contact‐independent transfer. We also use ‘mitochondrial transfer axis’ to denote defined donor–acceptor pairs observed *in vivo* and discuss evidence standards recommended for robust assignment of transfer events [16].

## Modes of horizontal mitochondrial transfer (HMT)

**Fig. 2:**
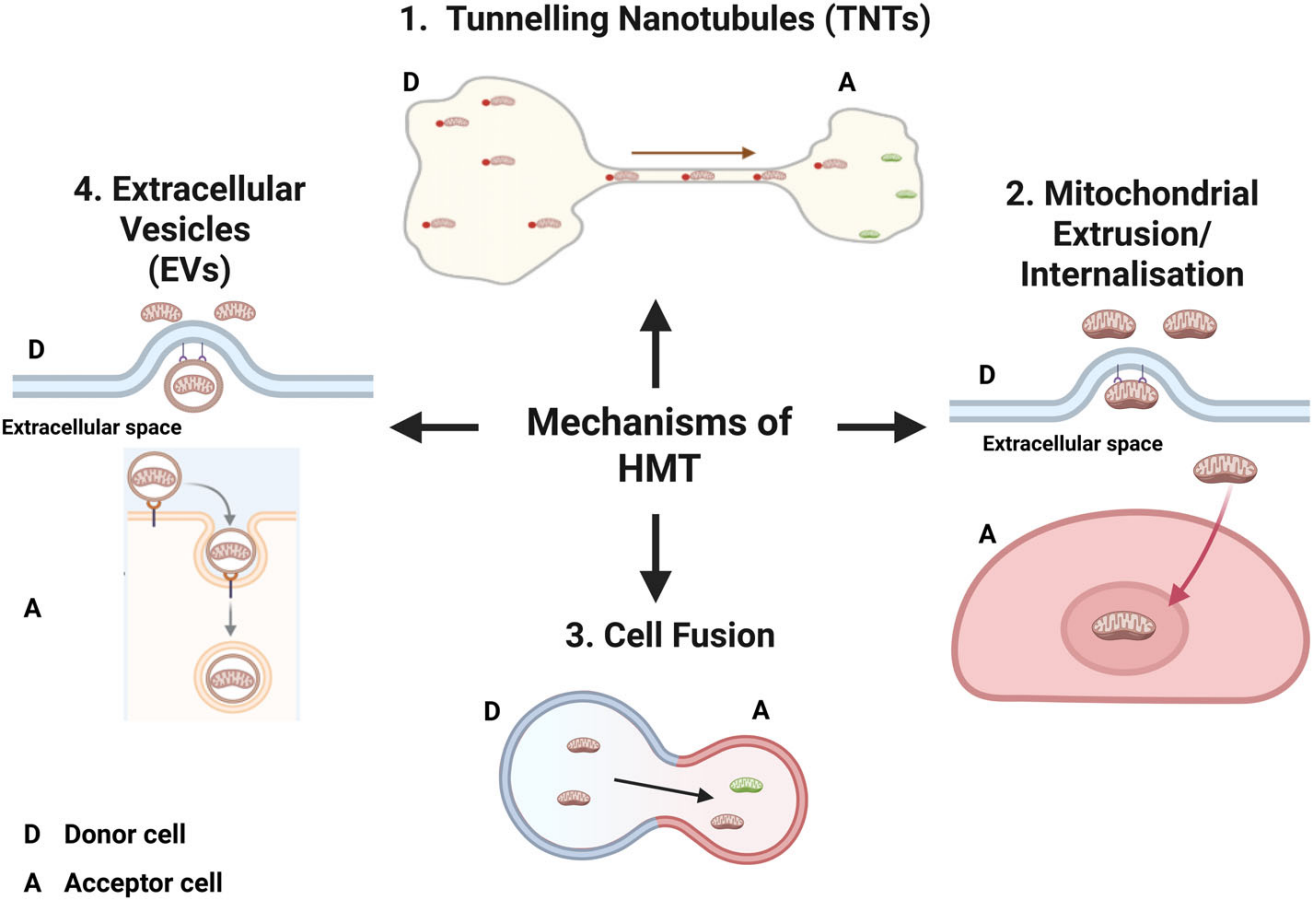
Mechanisms of horizontal mitochondrial transfer (HMT). Several mechanisms by which HMT occur have been identified to date including: (1) tunnelling nanotubules (TNTs), where the donor and acceptor cells are connected by nanoscale molecular tunnels through which mitochondria are passed from the donor to the acceptor cells. (2) mitochondrial extrusion/internalisation, where donor cells extrude naked mitochondria in the extracellular space which are taken up by the acceptor cells via macropinocytosis. (3) cell fusion, where two neighbouring cells fuse their cytoplasm and exchange cytoplasmic contents directly, including mitochondria. (4) Extracellular vesicles, where mitochondria are released within enclosed vesicles and taken up by the acceptor cells via receptor‐mediated endocytosis. Created in BioRender. Nwarunma, E. (2026) https://BioRender.com/nozauuk. Licence agreement number: SS29IK1ZAE.

HMT is a form of intercellular communication in which functional mitochondria are shuttled from donor to acceptor cells, enabling metabolic adaptation and stress resistance. Distinct modes of transfer have been identified, as shown in Fig. 2, with direct transfer mediated via TNTs being the most studied and characterized, both under steady‐state physiological conditions, such as repair, and pathophysiological conditions such as cancer [17, 18, 19]. Other direct modes of HMT include mitochondrial extrusion/internalization, gap junctions (GJs) [11], and cell fusion. Conversely, indirect mechanisms of HMT include uptake of mitochondria‐containing extracellular vesicles (EVs) [20, 21].

## TNTs

TNTs, the most robustly studied mode of HMT in both physiological and pathophysiological conditions, are thin filamentous‐actin (F‐actin)‐rich membranous bridges connecting cells over distances typically ranging from 50 nm to >1 μm and can have diameters within the range of 50 nm to 700 nm that facilitate transfer of organelles, including mitochondria, along cytoskeletal tracks [10, 22]. TNTs can be either open‐ended (direct cytoplasmic continuity) or closed‐ended (with gap junction caps) and may form through membrane protrusions or cell dislodgement mechanisms [23]. Molecular drivers include Leukocyte‐Specific Transcript 1 (LST1), M‐sec (Tumour Necrosis Factor Alpha Induced Protein 2 [TNFAIP2]), RAS Like Proto‐Oncogene A (RalA), and the exocyst complex, which coordinate actin polymerization and membrane protrusion formation [23]. It has also been reported that activation of the Akt–mTOR axis under stress (e.g., via chemotherapy or ROS exposure) enhances TNT biogenesis [23]. The mitochondrial adaptor protein Miro1 facilitates organelle motility along TNTs, linking mitochondria to the motor complex and supporting directed transfer [10, 24]. Also, myosin proteins, specifically Myo10, have been described as a positive regulator of TNT. As a cargo transporter, it is suggested to deliver the necessary actin regulators to the growing tip of the TNTs [25]. Functionally, TNT‐mediated HMT has been demonstrated across diverse cell systems. For example, mitochondrial rescue, via TNTs, was shown between MSCs and solid cancers, restoring respiration in mitochondrial DNA (mtDNA)‐depleted tumour cells [10, 24]. In cancer models including AML and MM, TNTs enable stromal‐to‐tumour cell mitochondrial import, bolstering OXPHOS and chemoresistance [23, 24].

## Extracellular vesicles (EVs) and mitochondrial extrusion

Some cells release EVs containing mitochondria or as free organelles, which can be internalized by acceptor cells. EV‐mediated HMT has been described in cardiac, neural, and epithelial contexts. For instance, mitochondrial extrusion occurs via mitotic bodies or membrane blebs that release fragmented mitochondria for uptake by neighbouring cells [11]. Although EV‐mediated transfer has been suggested in cancer and MSC systems [5], it is less frequently reported than TNT‐mediated mechanisms and has been more challenging to quantify both *in vitro* and *in vivo* [24]. It has also been reported in a recent nature paper that cancer cells steal mitochondria from T‐cells via small EVs rather than micro EVs [5].

## Gap junction channels (GJs)

Gap junctions formed by connexin proteins (such as Connexin‐43/Cx43) connect adjacent cell cytoplasms and allow passage of small molecules, ions, and second messengers (< 1 kDa). Although their channels are too narrow for whole mitochondria, GJs may support TNT formation and indirectly facilitate mitochondrial transfer [26]. In MSC‐tumour models, Cx43 expression was required for efficient mitochondrial delivery, suggesting a regulatory role in initiating TNT formation or vesicular exchange [27].

## Cell fusion

Cell fusion allows direct sharing of cytoplasmic contents, including mitochondria, between two cells. While this phenomenon has been rarely observed within *in vivo* cancer models, partial or transient fusion events, sometimes overlapping with TNT formation, have been described in MSC‐epithelial or stem cell co‐cultures, enabling mitochondrial exchange and subsequent metabolic reprogramming of the acceptor/epithelial cell [11].

## Directionality of HMT

Studies have reported TNT‐mediated HMT as unidirectional, Lu J and colleagues reported unidirectional transfer of mitochondria from more invasive T24 bladder cancer cells to less invasive RT4 bladder cancer cells, making them more invasive [10]. Intercellular mitochondrial transfer is often depicted as a unidirectional process driven by injury or stress, yet compelling evidence now demonstrates that under certain physiological and pathological contexts, mitochondrial exchange can be bidirectional. One of the earliest studies to report this phenomenon was conducted by Korpershoek *et al*. (2022), who investigated co‐cultures of chondrocytes and MSCs. They detected mitochondria originating from chondrocytes entering MSCs, and vice versa, via mechanisms including TNTs, EVs and direct cell‐to‐cell contact, marking a clear case for bidirectional organelle trafficking [28]. In the immune compartment, Somova *et al*. studied interactions between MSCs and human B lymphocytes, finding that MSCs transferred healthy mitochondria to activated B cells, which lead to enhanced viability and proliferation in the activated B cells. Simultaneously, dysfunctional mitochondria were passed from immune cells to MSCs, indicating a mutual exchange that may underlie immunomodulatory processes [29]. A landmark study by Ikeda *et al*. (2025) in *Nature* established bidirectional HMT within the tumour microenvironment, revealing how cancer cells manipulate immune effector mechanisms to evade elimination. The authors analysed clinical specimens from patients with melanoma and non‐small‐cell lung cancer. They discovered that mutated mitochondrial DNA (mtDNA) from cancer cells is transferred into tumour‐infiltrating lymphocytes (TILs). This transfer leads to the replacement of healthy mtDNA in TILs with cancer‐derived copies (i.e., homoplasmy), which in turn impairs mitophagy, the autophagic degradation of damaged or superfluous and mitochondrial fission and undermines T‐cell metabolic fitness and effector function. Immunologically, the acceptor TILs exhibit hallmarks of senescence, having increased expression of senescence molecules p16 and p53. They also showed hallmarks of metabolic dysfunction, reduced proliferative capacity, diminished activation marker expression, such as PD‐1 and CD69, and impaired memory formation. Overall, this resulted in compromised anti‐tumour immunity and poor responses to immune checkpoint inhibitors [5].

## Molecular triggers of HMT

Intercellular mitochondrial transfer is typically initiated by cellular stress, including elevated ROS, chemotherapy, hypoxia and metabolic dysfunction [30]. Under physiological conditions, these stress signals enable donor cells to support compromised acceptor cells and maintain tissue homeostasis. For instance, Mistry *et al*. showed mitochondrial transfer from BMSCs to HSCs in response to bacterial infection triggered by ROS and mediated by PI3K activation [31]. In haematological malignancies, malignant cells appear to hijack these conserved stress‐response pathways to reinforce survival. Tumour‐derived ROS driven by daunorubicin (DNR) as well as hypoxia (induced by CoCl_2_) have been shown to induce TNT formation in stromal cells via NADPH oxidase 2 (NOX2), facilitating mitochondrial transfer to AML blasts [32]. Similarly, the cell surface membrane glycoprotein CD38, which can act as both an enzyme and receptor, promotes TNT formation and mitochondrial trafficking. It achieves this by regulating calcium signalling via its ecto‐enzymatic metabolism of NAD^+^, which drives cytoskeletal rearrangements necessary for TNT extension [32].

## HMT in acute myeloid leukaemia (AML)

HMT has surfaced as a pivotal metabolic adaptation employed by blood cancer cells within the bone marrow niche, enabling malignant cells to withstand chemotherapeutic stress and persist in hostile microenvironments. In AML, pioneering work by Moschoi *et al*. (2016) revealed that leukaemic blasts internalize mitochondria from BMSCs, via cell‐to‐cell contact and endocytic mechanisms. This transfer led to increased mitochondrial mass by approximately 14%, resulting in an increase in OXPHOS‐derived ATP production by approx. 1.5‐fold. Importantly HMT conferred resistance to cytarabine (Ara‐C) as shown within an *in vitro* setting utilizing BMSC‐AML co‐culture systems and *in vivo* using the MOLM‐14 xenograft mouse model [14]. Expanding on this, Marlein *et al*. demonstrated that AML‐derived ROS, generated by NOX2, serves as the proximate trigger for HMT. ROS were shown to stimulate TNT formation between BMSCs and AML blasts. shRNA‐mediated knockdown or pharmacological inhibition of NOX2 (using diphenyleneiodonium [DPI]), as well as disruption of actin polymerization, markedly curtailed HMT, increased apoptosis in AML blasts, and extended survival in patient‐derived xenograft (PDX) and OCI‐AML3‐luc xenograft models [32]. In their 2023 *Science Advances* paper, Weinhäuser and colleagues revealed that M2‐polarized macrophages in the AML BMME bolster leukaemic transformation by transferring mitochondria to AML blasts. This mitochondrial exchange culminates in enhanced OXPHOS and metabolic fitness in the blasts. Importantly, blasts co‐cultured with M2 macrophages displayed reduced phagocytosis, enhanced mitochondrial metabolism via direct HMT, as demonstrated by mitochondrial labelling and flow cytometric tracking. This study therefore highlighted a key role for metabolic crosstalk in leukaemic aggressiveness and niche/macrophage‐driven evolution of AML clones [6]. Further mechanistic insight stems from studies linking stromal priming to mitochondrial biogenesis. Marlein *et al*. later reported that AML cells upregulate peroxisome proliferator‐activated receptor gamma coactivator 1‐alpha (PGC‐1α) in BMSCs. This enhanced mitochondrial generation in the stromal cells, thereby ensuring a replenishable mitochondrial reservoir for HMT to AML cells [32].

## Mitochondrial transfer in multiple myeloma (MM)

In the context of MM, HMT research is more nascent, but equally as compelling. Matula *et al*. (2023) explored mitochondrial interactions in primary patient‐derived MM‐BMSC co‐cultures treated with belantamab mafodotin, a BCMA‐targeted antibody‐drug conjugate. They discovered bidirectional HMT. However, surviving MM cells disproportionately acquired stromal mitochondria under drug‐induced stress. These drug‐tolerant/surviving cells demonstrated elevated mitochondrial uptake, enhanced metabolic activity, and increased survival, suggesting that mitochondrial acquisition serves as a compensatory survival mechanism in response to cytotoxic therapy [33]. In another study, Marlein *et al*. (2019) reported the involvement of CD38 in mitochondrial transfer from BMSC to MM cells *in vitro* and *in vivo* orchestrating metabolic plasticity [13]. Altogether, these primary studies illustrate that HMT represents a dynamic metabolic symbiosis, driven by drug‐induced and tumour‐intrinsic oxidative stress and enabled by stromal priming and structural coupling. AML and MM share conserved mitochondrial transfer mechanisms centred on TNTs, connexin‐mediated contacts, and HMT triggers such as oxidative stress, leading to metabolic enhancement and drug resistance. Also, they both receive mitochondria from the same cell types within the BMME, such as BMSCs. Since it has been shown that M2 macrophages transfer mitochondria to AML blasts, it is also tempting to speculate that MM cells could also receive mitochondria from M2 macrophages, as M2 macrophages are also found to be increased in the BM of MM patients [34, 35]. Studies have also demonstrated that macrophages drive therapy resistance in MM [36]. Their parallel dependence on BMME‐resident cells highlights HMT as a potential shared therapeutic vulnerability in AML and MM.

## HMT in other blood cancers

In B‐ALL, Burt and colleagues showed that chemotherapy (e.g., ROS‐inducing agents) activates mesenchymal stromal cells toward a cancer‐associated fibroblast‐like state that forms TNTs and transfers functional mitochondria to ALL cells, rescuing mitochondrial fitness and protecting leukaemic cells from oxidative stress‐driven apoptosis [15]. In lymphoid malignancies, TNT‐like nanotubes have also been implicated in direct mitochondrial trafficking between malignant B cell**s**. Halász *et al*. reported uni‐ and bidirectional mitochondrial transport through nanotubes in B‐lymphoma cells, driven by coordinated actin–microtubule cytoskeletal components and motor proteins, supporting the concept that mitochondrial exchange can occur within malignant lymphoid networks [37].

In chronic myeloid leukaemia (CML), stromal cells form TNTs that deliver prosurvival cargo (vesicles/proteins) and promote imatinib resistance; while this work does not establish mitochondrial transfer *per se*, it highlights that TNT‐based intercellular trafficking is operational in CML and may provide a structural route for organelle transfer under appropriate conditions [38]. Collectively, these studies support mitochondrial transfer as a broader feature of haematological malignancies, with particularly strong original evidence in B‐ALL and emerging mechanistic insight in lymphoid tumours.

## Mechanistic insights

Gap junction coupling via connexin‐43 supports transfer, and its inhibition reduces leukaemic stem cell clonogenicity [26]. Within acceptor blasts, donor uptake is coordinated with mitophagy, which clears damaged endogenous organelles and facilitates integration of healthier imported/transferred mitochondria. Strikingly, OXPHOS inhibitors can paradoxically stimulate this process, allowing blasts to escape metabolic blockade [39]. Pharmacological modulation of mitophagy, for example via p62/SQSTM1 or Hsp70‐Parkin pathways, alters AML survival and enhances chemosensitivity in AML cells [40]. Calcium signalling intersects these processes. While direct evidence in AML is lacking, studies in solid tumours show that Ca^2+^ flux through IP_3_ receptors and mitochondrial permeability transition pores regulates mitochondrial dynamics and mitophagy via CAMKII‐AMPK‐mTOR signalling [41]. Cytoskeletal regulators such as the RhoC‐LIMK axis, essential for filopodia extension in other cancers, are also plausible but still untested candidates for TNT regulation in AML [39]. Recent data from our lab indicate that the RhoC‐LIMK axis plays a key role in phosphorylation/deactivation of cofilin and subsequently mitochondrial transfer in AML [42]. Cofilin has been shown to play a central role in regulating actin cytoskeletal dynamics, which is essential for TNT formation and mitochondrial transfer [43].

In MM, CD38 expression drives TNT formation and mitochondrial acquisition from BMSCs, conferring OXPHOS plasticity; CD38 inhibition reduces transfer and metabolic fitness [13]. Stress induced by proteasome inhibitors such as bortezomib enhances mitochondrial uptake in surviving MM cells, suggesting a compensatory mechanism akin to AML [44]. Beyond organelle trafficking, mtDNA transfer has functional significance.

Donor mtDNA can restore respiration in cells with compromised genomes and may create heteroplasmy, enhancing metabolic adaptability and clonal evolution. In other tumour contexts, horizontal mtDNA transfer reinstates tumour‐initiating potential [39], and similar processes likely occur in AML and MM. Altered mtDNA also shapes nuclear transcription through retrograde signalling, influencing redox balance and epigenetic programming. Thus, mtDNA transfer acts not only as an energetic lifeline, but also as a driver of genetic and signalling reprogramming.

Together these observations suggest that HMT in AML and MM reflects a coordinated network of signals: ROS and Ca^2+^ flux trigger TNT formation; stromal cells respond with PGC‐1α‐driven biogenesis and connexin‐43 coupling; acceptors activate mitophagy and fission to integrate donor mitochondria; and mtDNA exchange enhances metabolic flexibility. Targeting these axes, via NOX2 inhibition, metformin, TNT disruptors or mitophagy modulators, offers opportunities to disrupt tumour–stroma metabolic symbiosis. The summary of the mechanistic insights is provided in Table 1.

**Table 1:** Summary of the mechanistic insights of mitochondrial transfer in AML and MM.

Mechanism	Molecular initiators/regulators	Impact on AML / MM cells	Therapeutic implications
ROS (NOX2)	AML‐derived superoxide; activates PI3K‐Akt	Induces TNT formation, mitochondrial transfer in AML	NOX2 inhibitors [45]
PGC‐1α‐mediated biogenesis	Stromal metabolic reprogramming	Increases donor mitochondria pool in AML	PGC‐1α inhibitor to prevent stromal preparedness [46]
Connexin‐43 (Cx43)	Gap junction‐mediated adhesion	Enhances stromal–LSC coupling and HMT in AML	Cx43 inhibitors [26]
Mitophagy & fission	PINK1–Parkin, p62 pathways, fission enzymes	Removes damaged mitochondria, integrates donor mitochondria	Mitophagy inhibitors (e.g., XRK3F2), Hsp70 modulators [47]
Calcium/cAMKII–AMPK/mTOR	ER–mitochondrial Ca^2+^ flux, apoptosis/mitophagy regulation	Influences mitochondrial dynamics, stress response	Calcium modulators, IP_3_R blockers (preclinical) [41]
RhoC–LIMK cytoskeletal axis	Actin polymerisation required for TNT formation	Potential regulator of TNT biogenesis, potentially in AML and MM	LIMK inhibitors [39]
Metformin	Inhibits direct HMT	Reduces OXPHOS and sensitizes AML cells to Ara‐C	Repurposing in AML therapy [48]
mtDNA Transfer	Horizontal mtDNA exchange via TNTs or vesicles	Restores respiration and tumour initiation in AML or MM	Strategies to prevent mtDNA uptake [39]

## Functional consequences of HMT in haematological malignancies

### Enhanced metabolic capacity

HMT fundamentally remodels the metabolic circuitry of haematological malignancies, via impacts on OXPHOS and redox homeostasis in acceptor tumour cells. In AML, TNT‐mediated HMT from BMSCs has been shown to directly augment leukaemic blast respiration and ATP generation. Moschoi *et al*. first demonstrated that stromal‐derived mitochondria enhanced OXPHOS and conferred resistance to ara‐C by sustaining mitochondrial membrane potential and reducing apoptosis [14]. Subsequently, Marlein *et al*. confirmed that AML cells induce NOX2‐dependent ROS to trigger TNT formation and mitochondrial uptake, which significantly increases oxygen consumption and energy production [32]. Enhanced OXPHOS enables blasts to maintain ATP levels during glucose restriction, positioning high‐OXPHOS as both adaptive and therapeutically vulnerable states [26, 49, 50]. Stromal‐derived mitochondria not only enhance respiration but also accelerate AML proliferation [6, 49]. Similar observations have been made in MM, where Marlein *et al*. identified CD38‐mediated TNT formation as a driver of mitochondrial trafficking from BMSCs to myeloma cells, promoting a shift from glycolysis toward OXPHOS and enhancing ATP output and drug resistance [13]. Blocking this transfer, either by disrupting TNTs or inhibiting CD38, reduces mitochondrial respiration and sensitizes tumour cells to chemotherapeutics. Collectively, these findings establish HMT as a key mechanism of metabolic augmentation, enabling AML and MM cells to transcend intrinsic mitochondrial defects and sustain the high energy demands of proliferation, survival and relapse.

### Redox regulation and ROS homeostasis

Beyond metabolic enhancement, HMT plays a crucial role in maintaining redox equilibrium within malignant haematopoietic cells. AML and MM cells experience high oxidative stress due to rapid proliferation and dysregulated mitochondrial metabolism, yet they rely on a finely tuned redox balance to prevent apoptosis. By acquiring healthy mitochondria from BM resident cells, these tumour cells replenish their antioxidant capacity and stabilize ROS levels.

Marlein *et al*. demonstrated that AML blasts with elevated NOX2‐derived superoxide actively stimulate TNT formation, acquiring mitochondria that restore redox homeostasis and prevent ROS‐induced cell death [32]. Similarly, Moschoi *et al*. showed that mitochondria transferred from BMSCs to AML blasts suppress intracellular ROS accumulation and enhance mitochondrial integrity, thereby conferring resistance to oxidative stress and chemotherapy [14]. In MM, Marlein *et al*. found that CD38‐driven mitochondrial acquisition improves redox buffering through enhanced NADPH generation and OXPHOS flux, supporting cell survival under proteasome inhibitor stress [13]. It is tempting to then speculate that inhibition of HMT or OXPHOS would lead to excessive ROS accumulation, mitochondrial depolarization and apoptosis, underscoring the dependency of malignant cells on redox regulation via stromal mitochondria. In line with these observations, our recent findings show that DNR exposure induces a robust increase in ROS generation in AML cells, an effect substantially diminished by co‐culture with M2 macrophages. [42]. These findings highlight HMT as a redox‐protective mechanism, coupling metabolic resilience with antioxidant defence, to enable sustained growth and therapy resistance in the oxidative microenvironment of the BMME.

### Chemoresistance

Mechanistically, leukaemic stem cells (LSCs) display remarkable plasticity. Under OXPHOS inhibition or ara‐C induced stress, LSCs use TNTs to acquire new mitochondria while discarding damaged ones via fission and mitophagy, thereby surviving and acquiring secondary resistance [39]. Ara‐C resistant AML cells exhibited increased mitochondrial mass and ROS, with metabolic gene signatures indicating heavy metabolic reliance on mitochondrial oxidative phosphorylation. AML blasts also rewire energy‐sensing pathways. Stromal‐induced OXPHOS activation suppresses AMPK and sustains mTORC1, reinforcing anabolic growth and resistance. Pharmacological restoration of AMPK activity reverses this phenotype and suppresses disease progression *in vivo* [12, 45]. In parallel, HMT augments leukaemic stemness: enforced connexin‐43 expression in BMSCs increases HMT to LSCs, enhancing adhesion, proliferation, clonogenicity, and a stemness‐related gene signature, while reprogramming central metabolic pathways [26]. In MM, CD38‐mediated mitochondrial uptake from BMSCs drives a metabolic shift toward OXPHOS, increasing ATP production and survival, particularly under chemotherapeutic stress. Blocking TNTs with cytochalasin B reduces transfer and restores drug sensitivity in both MM and AML contexts [13, 42]. Moreover, transferred mitochondria bring their mtDNA, which restores respiratory competence and creates heteroplasmy, enhancing clonal adaptability. This horizontal mtDNA transfer, observed across tumour models, likely contributes to AML and MM metabolic resilience in the bone marrow niche [39]. Emerging evidence from solid tumours also suggests HMT into T cells can impair immunity through mutated mtDNA and defective mitophagy. While not yet demonstrated in haematological malignancies, such crosstalk may contribute to immune evasion within the BMME in AML and MM.

To accommodate the increased mitochondrial load that accompanies HMT, AML and MM cells appear to engage active mitochondrial quality control and disposal programs. In AML, Saito *et al*. showed that under cytarabine or OXPHOS‐inhibitory stress, leukaemic cells couple mitochondrial acquisition with mitochondrial fission and mitophagy, selectively removing damaged endogenous organelles to ‘make room’ for newly acquired functional mitochondria and thereby sustain bioenergetic fitness and drug tolerance [39]. Complementing this intracellular route, Solimando *et al*. reported that in myeloma, tumour cells can export dysfunctional mitochondria to BMSCs via TNTs for lysosomal degradation, consistent with transmitophagy, which reduces mitochondrial stress and supports resistance to apoptosis under metabolic challenge [51]. Together, these findings suggest that malignant cells not only enhance mitochondrial acquisition but also actively manage mitochondrial excess through coordinated intracellular turnover and intercellular offloading. The functional consequences are highlighted in Table 2.

**Table 2:** Functional consequences of mitochondrial transfer and underlying mechanisms.

Functional consequence	Underlying mechanism(s)	Observed effect in AML / MM
OXPHOS dependency	Transfer of functional mitochondria via TNTs; enhanced Complex I	Sustained ATP production during glucose inhibition or chemotherapy stress in AML or MM [50].
Chemoresistance	mtDNA restoration, mitophagy + fission, metabolic reprogramming	Resistance to Ara‐C, OXPHOS inhibitors, and venetoclax [39]
AMP‐AKT/mTOR modulation	Stromal contact‐induced ATP/metabolic signalling alterations	AMPK suppression: mTORC1 activation promotes survival under stress [12, 45].
Enhanced stemness	Cx43‐mediated transfer promotes metabolic and transcriptional shifts	Greater adhesion, clonogenicity, and LSC survival [26].
Metabolic plasticity in MM	CD38‐driven TNT formation and donor mitochondrial incorporation	Glycolysis‐to‐OXPHOS switch supports survival during chemotherapy [13].
**m**tDNA restoration	Horizontal mitochondrial and mtDNA transfer	Recovery of respiratory competence after mtDNA damage [39].
Potential immune evasion	Aberrant mtDNA transfer and mitophagy impairment in immune cells	T cell dysfunction via HMT [5].

### Therapeutic targeting of HMT in haematological malignancies

Disrupting HMT between BMME‐resident stromal and immune cells, such as BMSCs and macrophages and blood cancer cells, offers a promising therapeutic strategy to overcome BMME‐driven therapy resistance. Some molecular nodes, including CD38, NOX2, mitochondrial metabolism and the RhoC‐LIMK1/2‐Cofilin pathway, have emerged as actionable targets, with preclinical studies highlighting their therapeutic potential. One particularly plausible therapeutic approach is targeting CD38, with known CD38‐targeting therapies (e.g., Daratumumab) already in clinical use for the treatment of MM. Beyond its many immunomodulatory roles, CD38 regulates TNT formation and HMT in MM. In MM, CD38 knockdown reduced TNT frequency, reduced mitochondrial uptake by 50%, impaired OXPHOS, and prolonged survival in murine models of myeloma [13]. In AML, daratumumab inhibited stromal‐to‐blast HMT *in vitro* and in AML xenografts, reducing mitochondrial mass, membrane potential, and oxygen consumption in blasts, resulting in hindering disease progression. Notably, these effects were independent of immune effector activity, underscoring the important non‐immunological role for CD38 in metabolic crosstalk [52, 53]. A second potential therapeutic opportunity is targeting NOX2. Although no NOX2 inhibitors are currently in clinical use or being tested in clinical trials, NOX2 inhibition has shown potential in preclinical contexts. Inhibition of NOX2‐derived ROS, using the pan‐NOX inhibitor DPI, markedly reduced TNT formation and stromal‐to‐blast mitochondrial trafficking, thereby restoring chemosensitivity and prolonging survival in patient‐derived blasts [32]. More selective next‐generation NOX2 inhibitors, including VAS2870, GSK2795039 and indole‐based derivatives, are currently under preclinical evaluation for their ability to limit oxidative stress‐driven mitochondrial trafficking and metabolic reprogramming in AML [54]. Targeting NOX2, particularly when combined with glycolytic blockade (e.g., using VAS2870), has shown strong synergy: dual inhibition suppressed AML proliferation, reduced clonogenicity, impaired chemoresistance and delayed disease onset in murine models of AML [54].

These findings suggest that interfering with oxidative stress‐induced HMT may enhance the efficacy of conventional therapies. Metformin, a biguanide antihyperglycemic agent clinically approved for the management of type II diabetes, has also gained prominence for its dual effects on AML metabolism and HMT. Metformin has been shown to increase survival in FLT‐3 positive AML [55] and its combination with venetoclax and ara‐C is currently in phase 2 clinical trial (NCT06537843) for the treatment of relapsed‐refractory, induction‐ineligible AML (https://clinicaltrials.gov/). In BMSC‐AML co‐culture systems, metformin reduced mitochondrial uptake, suppressed OXPHOS, and enhanced the efficacy of ara‐C in AML xenografts [56]. Mechanistically, metformin inhibits complex I, increases ROS, and sensitizes MLL/AF9‐driven AML cells to BCL2 antagonists, such as venetoclax [54, 57]. Metformin also potentially interferes with actin dynamics by modulating PP2A‐cofilin signalling, thereby impairing TNT formation [48]. Recent data from our laboratory show that metformin also inhibits HMT from M2 macrophages to AML cells, resensitizing AML cells to DNR‐induced apoptosis. These multifaceted actions highlight metformin's potential as a repurposed agent targeting both OXPHOS and HMT. At a broader level, combinatorial metabolic blockade, for instance, pairing NOX2 inhibitors with glycolysis or fatty acid oxidation inhibitors, has shown promise in targeting residual AML populations that rely on stroma cell‐derived mitochondria for persistence [54, 58]. It is possible that these combinatorial strategies will be particularly effective against minimal residual disease, where HMT supports relapse.

In summary, therapeutic targeting of mitochondrial transfer, via CD38 blockade, NOX2 inhibition, or biguanides such as metformin, offers a rational therapeutic avenue to weaken the metabolic symbiosis between tumour cells and the bone marrow niche. These approaches not only impair mitochondrial support but also have the potential to re‐sensitize blood cancer cells to chemotherapy, providing a translational framework for clinical development.

## Current challenges and future directions

Despite growing evidence that HMT can drive therapy resistance and metabolic adaptation in AML, MM and other haematological malignancies, translation of HMT research into clinical benefit faces a number of challenges. A key barrier is the intrinsic complexity of mitochondrial biology. Mitochondria differ in membrane potential, mtDNA copy number and respiratory chain activity both across cell types and within malignant clones. This heterogeneity likely influences how dependent/reliant blood cancer cells are on the acquisition of exogenous mitochondria, and how they respond to metabolic targeting. It has been shown that AML with MLL/AF9 rearrangement displays elevated mitochondrial mass and oxidative metabolism through NRF1 upregulation, rendering these cells particularly sensitive to OXPHOS inhibitors, such as metformin [57]. Mechanistic uncertainty complicates therapeutic development.

While TNTs are the most consistently observed conduits between AML/MM and donor bone marrow‐resident cells, mitochondria can also be exchanged via EVs, gap junctions, or as free organelles. Therefore, it remains unclear whether these pathways reflect a dedicated machinery or opportunistic adaptations of existing intercellular communication systems [59]. A related, but underexplored process, is transmitophagy, in which cells expel dysfunctional mitochondria for degradation by neighbouring stromal or immune cells [51]. This ‘reverse transfer’, observed in the MM context by Solimando *et al*. (2025), may also potentially enable AML or MM cells to preserve mitochondrial fitness. Another major challenge is balancing therapeutic specificity with preservation of physiological functions. ROS‐driven HMT and, in particular, TNT‐driven HMT play a role in tissue repair and haematopoietic stem cell (HSC) recovery. Thus, broad inhibition of regulators such as NOX2 may carry some risks. Although NOX2 inhibition reduces HMT and prolongs survival in AML xenograft models without impairing CD34^+^ progenitors [32], available inhibitors lack sufficient specificity to ensure safety in patients. Technical limitations in detecting and quantifying HMT also restrict progress.

Most current methods, such as labelling donor mitochondria with MitoTracker dyes, assess mitochondria based on their presence in acceptor cells by microscopy or flow cytometry. However, these methods capture only a snapshot of transferred mitochondria and cannot distinguish acceptors from non‐acceptors or track long‐term events [60]. Dye leakage observed with MitoTracker dyes adds additional complexities to data interpretation [60]. Alternative approaches, such as cross‐species co‐cultures detecting donor mtDNA, also lack temporal resolution. Recently, Hoover and colleagues introduced MitoTRACER, a method that permanently labels acceptor cells after acquiring donor mitochondria, enabling durable tracking of mitochondrial transfer dynamics [60]. Application of such tools to AML and MM is likely to refine mechanistic understanding. Pharmacological targeting of HMT remains at an early stage. Anti‐CD38 antibodies (e.g., daratumumab) block TNT formation and HMT, impairing metabolic support within preclinical models of AML and MM [53]. Metformin has shown efficacy in suppressing OXPHOS and HMT, resensitizing AML blasts to ara‐C, although its precise effects on stromal‐tumour crosstalk remain incompletely defined [56]. Other targets, including connexin‐43, remain experimental, and few inhibitors are clinically validated. Future work should prioritize patient stratification through advanced imaging and single cell profiling to identify subgroups dependent on stromal and immune‐mediated mitochondrial support. Given that AML and MM are predominantly age‐associated malignancies [61], it is plausible that HMT represents an ageing‐associated compensatory adaptation within the bone marrow niche, emerging in response to cumulative mitochondrial dysfunction, chronic inflammation, and impaired tissue repair. In this context, malignant cells may exploit a physiological stress‐response programme normally engaged to preserve haematopoietic fitness in aged marrow.

PDX and humanized bone marrow models will be crucial for optimizing therapeutic timing, such as introducing HMT inhibitors alongside chemotherapy or targeted therapies, such as venetoclax to prevent compensatory transfer at treatment onset. Modulating mitochondrial quality control, for example by inhibiting Dynamin‐like 120 kDa protein (OPA1)‐driven fusion or regulating mitophagy, also holds promise. Recent evidence shows that OPA1 inhibition impairs AML proliferation with limited effects on normal haematopoiesis [49]. Dissecting the pleiotropic effects of mitochondrial exchange, including heteroplasmy, retrograde signalling, and immune modulation, will be essential to understand its impact in full. The immunological dimension of HMT warrants particular attention. Studies in solid tumours indicate that tumour‐derived mitochondria or mtDNA can impair T‐cell function [5]. It is unknown whether similar mechanisms operate in AML and MM but targeting HMT may offer a dual benefit of disrupting tumour metabolism and restoring immune surveillance and immune‐mediated kill.

## Conclusion

In summary, HMT in AML and MM represents both a powerful adaptation and a therapeutic vulnerability. Key challenges include mitochondrial heterogeneity, incomplete mechanistic resolution, technical limitations in detection and risks of on‐target/off‐tumour toxicity. Addressing these issues will require more precise tools, more detailed in‐depth mechanistic studies, and careful translational design. Advances in metabolic imaging, gene editing technologies, and improved preclinical models provide a path forward, bringing the prospect of disrupting stromal/immune cell‐mediated HMT in AML and MM closer to clinical application.

## Authors contributions

EB and MTSW wrote the manuscript. EB prepared the figures and tables. EB and MTSW approved the submitted version.

## Conflict of interest

The authors declare no conflict of interest.
